# Nutritional status regulates algicidal activity of *Aeromonas* sp. L23 against cyanobacteria and green algae

**DOI:** 10.1371/journal.pone.0213370

**Published:** 2019-03-12

**Authors:** Susmita Das Nishu, Yunhee Kang, Il Han, Tae Young Jung, Tae Kwon Lee

**Affiliations:** Department of Environmental Engineering, Yonsei University, Wonju, Republic of Korea; INRA, FRANCE

## Abstract

Algicidal bacteria have received broad acceptance as an ecofriendly tool for controlling harmful algal blooms. However, their practical application is still limited to the lab-scale tests due to the complex alga–bacterium interactions in different nutrient statuses. In this study, the *Aeromonas* sp. L23 that exhibit relatively wide-spectrum in algicidal activity was isolated from a eutrophic agricultural lake. The physiological response of cyanobacteria and green to the algicidal activity under varied nutritional status were studied in an alga-bacterial co-culture. The algicidal activities of L23 against *Microcystis aeruginosa* UTEX LB 2385, *Microcystis aeruginosa* NHSB, *Anabaena variabilis* AG10064, *Scenedesmus quadricauda* AG10003, and *Chlorella vulgaris* AG10034 were 88 ± 1.2%, 94 ± 2.6%, 93 ± 0.5%, 82 ± 1.1%, and 47 ± 0.9%, respectively. The L23 cells had low algicidal activity in cell pellet (3%–9%) compared with the cell-free supernatant (78%–93%), indicating that the activity is induced by extracellular substances. Adding glucose, NaNO_3_, NH_4_Cl, and KH_2_PO_4_ to the co-culture raised the algicidal activity of the L23 against green algae by 5%–50%. Conversely, a 10%–20% decrease in activity occurred against the target cyanobacteria except *M*. *aeruginosa* UTEX LB 2385. These results indicated that the interspecific algicidal activity changes according to the nutritional status, which means that the alga-bacterium interaction will be more complex in the field where the nutritional status changes from time to time.

## Introduction

Harmful algal blooms (HABs) involve the rapid proliferation of phytoplankton, such as cyanobacteria and green algae, which produces toxins harmful to the environment [1]. Increases in HABs due to anthropogenic interference are not only damaging the environment [2] but also threatening water security and public health [1, 3].

The technologies for controlling HABs can be categorized into physical (e.g., clay and ultrasound) [4], chemical (e.g., copper sulfate, hydrogen peroxide, and potassium permanganate) [4], and biological controls (e.g., algicidal bacteria, fungi, and protozoa) [3]. The physical and chemical controls are effective but result in secondary pollution and are impractical for dealing with large-scale HABs observed in the environment. Therefore, biological controls for HABs, especially by using the natural algicidal bacteria, have attracted global attention for their economic efficiency, species-specificity, and eco-friendliness [3, 4].

Previous studies revealed algicidal activities of some genera including *Bacillus*, *Rhodococcus*, *Vibrio*, *Sphingomonas*, *Sphingosinicella*, *Actinobacter*, *Arthrobacter*, *Brevibacterium*, *Alcaligenes*, and *Sphingopyxis* which can control HABs in fresh water [5, 6]. Most of the widely acknowledged algicidal bacterial species belong to the phylum γ-Proteobacteria and Cytophaga–Flavobacterium–Bacteroides (CFB) [7]. Recently, several studies reported that some species of *Aeromonas*, including *A*. *hydrophila* [8, 9], *A*. *salmonicida*, *A*. *bivalvium* [10], and *A*. *caviae* [11], belong to the phylum γ-Proteobacteria can efficiently inhibit cyanobacterial growth by producing extracellular algicidal substances [7, 12]. However, most of the studies focused on the mode of attack or mechanism of algicidal bacteria under controlled conditions (temperature, irradiation, and light–dark cycle) [13].

The field application of algicidal bacteria is still in the early stage of development. While both the bacteria and the extracellular substances successfully controled single-species algal blooms in the laboratory conditions, this does not necessarily represent their applicability in the natural environment, which is more complex and variable [14]. In-situ mesocosm studies were performed to assess the risks and determine the ecological relevance of the laboratory-identified effects [13, 15]. Abiotic factors such as light, pH, nutrient flux, and temperature influence the interaction between algae and bacteria [13]. Among these factors, nutrient flux is the most important factor controlling the interactions of bacteria and algae by stimulating a change in survival strategy [16]. When algicidal species are present in the environment, they may not necessarily exhibit active anti-algal mechanisms as bacteria are capable of losing or switching off their metabolism [17]. Previous studies claimed that low-nitrogen conditions reduce the algal growth rate, subsequently increasing the intracellular lipid contents for energy storage [18]. It was also reported that the algicidal activity of algicidal strain *Pseudomonas fluorescens* SK09 is strongly associated with changes in nutritional status and consequent succession of phytoplankton species [15]. Despite the importance of nutritional status to the algicidal activity, information of algicidal activities on the different nutritional status is still lacking. This necessitates comprehensive studies on environmental conditions to develop an effective strategy to increase bacterial algicidal activity in HABs.

In this study, a novel algicidal bacterial strain was isolated from a freshwater lake and its algicidal activities against both cyanobacteria and green algae were characterized. The algicidal mode and antioxidative system in algal cells were studied to explore the algal inhibitory mechanism. Furthermore, the algicidal activities under different nutritional statuses were investigated to obtain a better understanding of the limiting factors in environmentally relevant conditions.

## Materials and methods

### Algal cultures

In this study, five algal strains were used as targets, including three cyanobacterial strains and two green algal strains. The three cyanobacterial strains were microcystin-producing *Microcystis aeruginosa* UTEX LB 2385 and microcystin non-producing strain NHSB (provided by Konkuk National University) and *Anabaena variabilis* AG10064 (purchased from Korean Collection for type cultures (KCTC)). The two green algal strains were *Scenedesmus quadricauda* AG10003 and *Chlorella vulgaris* AG10034 (purchased from KCTC). All algal species were cultivated in BG11 culture medium for 72 h at 25°C, 40 μmol photons m^−2^ s^−1^, with a 12 h:12 h light–dark cycle, as described previously [3]. We used the all algal species under exponential phase (empirically takes 72 hours) to obtain the culture showing highest activity.

### Isolation of algicidal bacteria

Water samples were collected from the surface (0–30 cm) of the Maiji Lake in Wonju, South Korea (31° 24′ N, 120° 13′ E), using a standard water sampler in August 2016. The collected water was transferred into sterile bottles in an icebox (4°C) and transported to the laboratory. The samples were diluted with sterile DI water, spread onto LB agar plates, and incubated in the dark at 30°C for 24 h. Bacterial strains were isolated by streaking bacterial colonies onto fresh LB agar plates. The isolation process was repeated twice for purity. A total of 36 bacterial strains were isolated and one colony from each plate was inoculated into 25 mL of LB medium and incubated in the dark at 30°C for 48 h with shaking speed of 150 rpm. After incubation, 2 mL (6x10^6^ cells mL^-1^) of each bacterial culture was transferred into a test flask containing 6 mL (1x10^7^ cells mL^-1^) of each algal strain in the exponential-growth phase. All of the treatments involved cultivation at 25°C and 40 μmol photons m^−2^ s^−1^ of light under 12 h:12 h light–dark cycles. Same amount of algal culture without addition of bacterial culture was used as control. 2 mL of LB or NA medium was added to algal culture instead of a bacterial strain as an additional control to measure algicidal effects by bacterial strains more accurately. The algicidal activity was measured by cell counting using an optical microscope (CX22LEDRFS1; Olympus Corporation, Japan). The cell suspensions were briefly sonicated to disperse aggregated cell clusters before applying them to microscope slides. The screening process was based on previous work by Li et al. (2014). The algicidal rate was calculated using the following formula:

$$\text{Algicidal}\;\text{rate}\;(\%)=(1-\frac{\text{DTt}}{\text{DCt}})\times 100$$ (1)

where DT is the algal density of the treatment group (cells mL^−1^), DC is the algal density of the control group (cells mL^−1^), and t is the time in days (d).

### Identification of algicidal bacteria

The algicidal bacterial strains in the liquid cultures were taxonomically identified using 16S rRNA gene analysis. The DNA was extracted from the liquid cultures using the FastDNA SPIN KIT (MP Biomedicals, USA), following the manufacturer’s instructions. The bacterial 16S rRNA gene was PCR-amplified with 27F (5′-AGAGTTTGATCMTGGCTCAG-3′) and 1492R (5′-GGHTACCTTGTTACGACTT-3′) primers [19]. The PCR reactions were performed in a total volume of 25 μL using TaKaRa Ex Taq (Takara Bio, Inc., Japan), in accordance with the manufacturer’s instructions. The PCR conditions were as follows: initial denaturation at 95°C for 4 min, followed by 30 cycles of denaturation at 95°C for 40 s, annealing at 54°C for 30 s, and extension at 72°C for 40 s. The PCR products were purified using a PCR purification kit (Qiagen, Germantown, USA), following the manufacturer’s instructions. Samples were sent to Macrogen Ltd. (Seoul, South Korea) for sequencing. Nucleotides were compared using the National Center for Biotechnology Information database (http://www.ncbi.nlm.nih.gov/BLAST). The Bioedit program was used for aligning the sequence and a phylogenetic tree was constructed by the neighbor-joining method using the software MEGA 5. The sequence of the 16S rRNA gene has been deposited in GenBank under accession number MH381782.

### Algicidal mode of bacterial strain

The direct and indirect algicidal modes of isolated bacterial strains were determined by inoculation of bacterial cells and cell-free suspension in algal growth experiments, respectively. The bacterial cells for the direct mode experiment was obtained by washing the culture using LB medium. The cultures (2 mL) were centrifuged at 10,000 × g for 10 min and the supernatants were discarded. The pellets were washed using fresh LB medium three times. The bacterial cell-free suspension for indirect mode experiment was collected by filtering supernatants with a 0.22-μm membrane filter (SP25P020SS; Hyundai Micro, South Korea). The bacterial cells and cell-free supernatant were added to 6 mL of algal cultures under the exponential-growth phase for indirect and direct mode tests, respectively. In addition, 2 mL of fresh LB medium was added to an algal culture as a control. Each treatment was replicated three times. The algicidal efficiency was calculated according to Eq (1).

### Antioxidative enzyme assays of algal cells

The algal culture treated with bacterial cells and cell free suspensions were collected every 24 h and tested by an antioxidative enzyme assay. For this, same amount (w) of cell pellet from control and each treatment were collected by transferring cultures to 2-mL sterilized tubes and centrifuged at 5,000 × g for 5 min. Supernatants were discarded and pellets were washed with PBS buffer three times. The washed cells were homogenized with ultrasonic cell pulverizer (Powersonic 603; Hwashin Technology Company) at 30°C for 30 min. The enzyme activities of superoxide dismutase (SOD), catalase (CAT), and peroxidase (POD) were measured using OxiSelect Superoxide Dismutase Activity Assay (Cell Biolabs Inc., USA), CAT100 (Sigma-Aldrich, USA), and OxiSelect Hydrogen Peroxide/Peroxidase Assay Kit (Colorimetric) (Cell Biolabs Inc., USA), respectively. All of the analytical methods followed the kit’s operation manual and absorbance values at different wavelengths were taken using a TECAN Spark 10M spectrophotometer (Männedorf, Zurich, Switzerland).

### Effect of medium on algicidal activity

Algicidal bacteria isolated from the water sample were inoculated into two different liquid media, namely, Luria–Bertani (LB) broth (244620—BD Difco, USA) and nutrient broth (NB) (234000—BD Difco, Sigma-Aldrich, USA), followed by incubation at 30°C for 48 h. After 5 days of incubation, the algicidal activities produced in the two media were compared to each other. The same amount of both media instead of a bacterial strain was inoculated into the algal culture as an additional control to measure the algicidal effects from media itself.

### Algicidal activity response to nutritional change

To investigate the change in algicidal activity according to different nutritional conditions, the sources of carbon, nitrogen, and phosphate were added to algal-bacterial mixture. A 2 mL (6x10^6^ cells/ml) bacterial culture was inoculated into 6 mL (1x10^7^ cells/ml) of each algal cultures supplemented with 1% and 2% (v/v) glucose, NaNO_3_, NH_4_Cl, and KH_2_PO_4_ and incubated for 5 days [1]. Samples were collected every 6 h to observe the growth rate of the bacterial strain in different nutrient conditions. Algicidal activity of the bacterial culture grown in LB medium without any supplement was used as a control. Three samples of each treatment were used to investigate the algicidal rate against all tested algal strains.

### Statistics

All data were analyzed using t-tests and ANOVA implemented in R (R Core Team, 2015). Data are presented as triplicate mean ± standard deviation of the mean and were evaluated using one-way analysis of variance followed by the least significant difference test, with p < 0.01 (**) and p < 0.05 (*).

## Result and discussion

### Isolation and identification of algicidal bacteria

**Fig 1:**
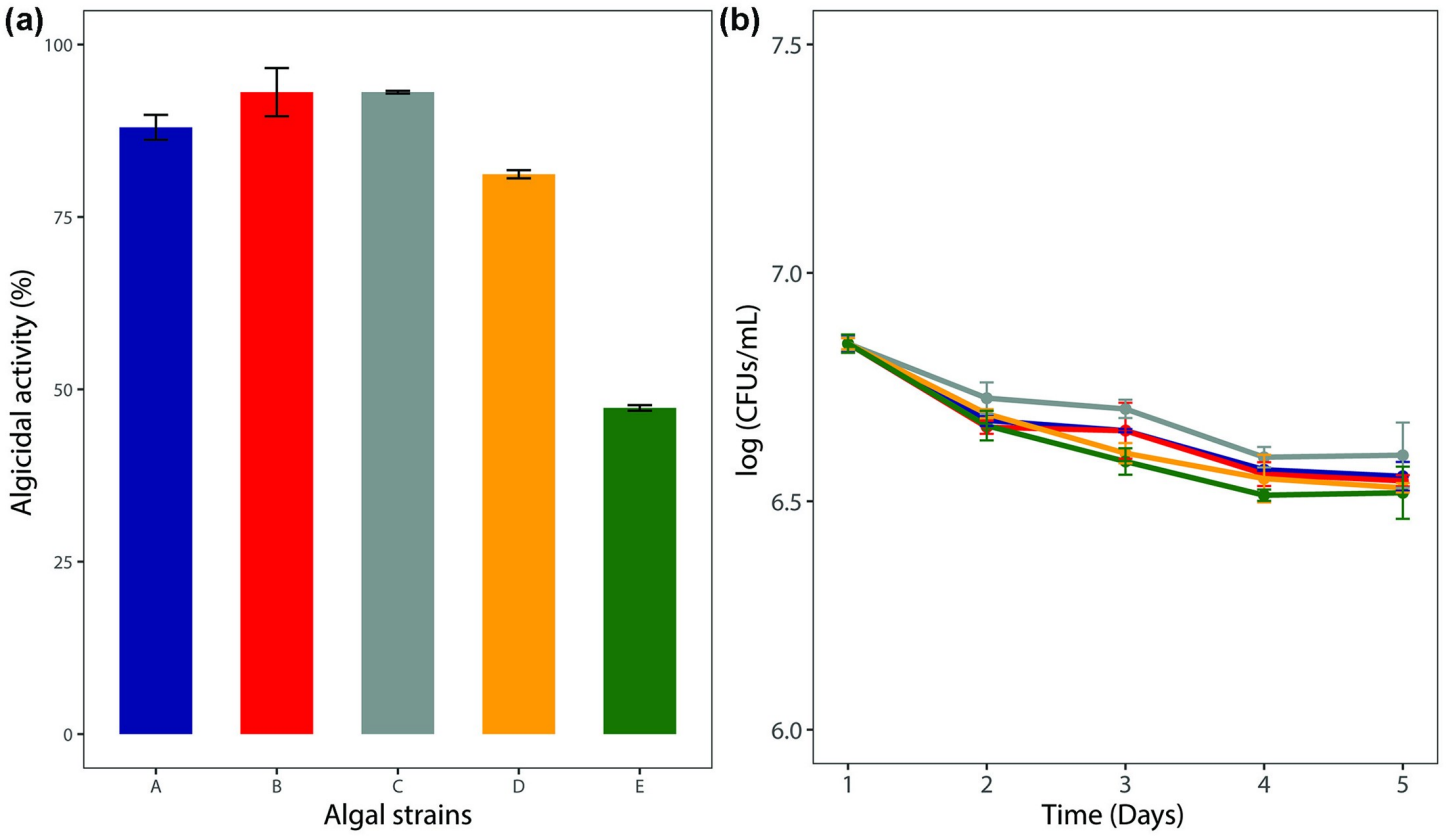
Algicidal activity of the strain L23. Algicidal activity against (A) *Microcystis aeruginosa* UTEX LB 2385, (B) *Microcystis aeruginosa* NHSB, [23] (C) *Anabaena variabilis* AG10064, (D) *Scenedesmas quadricauda* AG10003, (E) *Chlorella vulgaris* AG10034 after five days of treatment (a). Growth curve of the *Aeromonas* sp. L23 in the process of algicidal activity with time (b).

**Fig 2:**
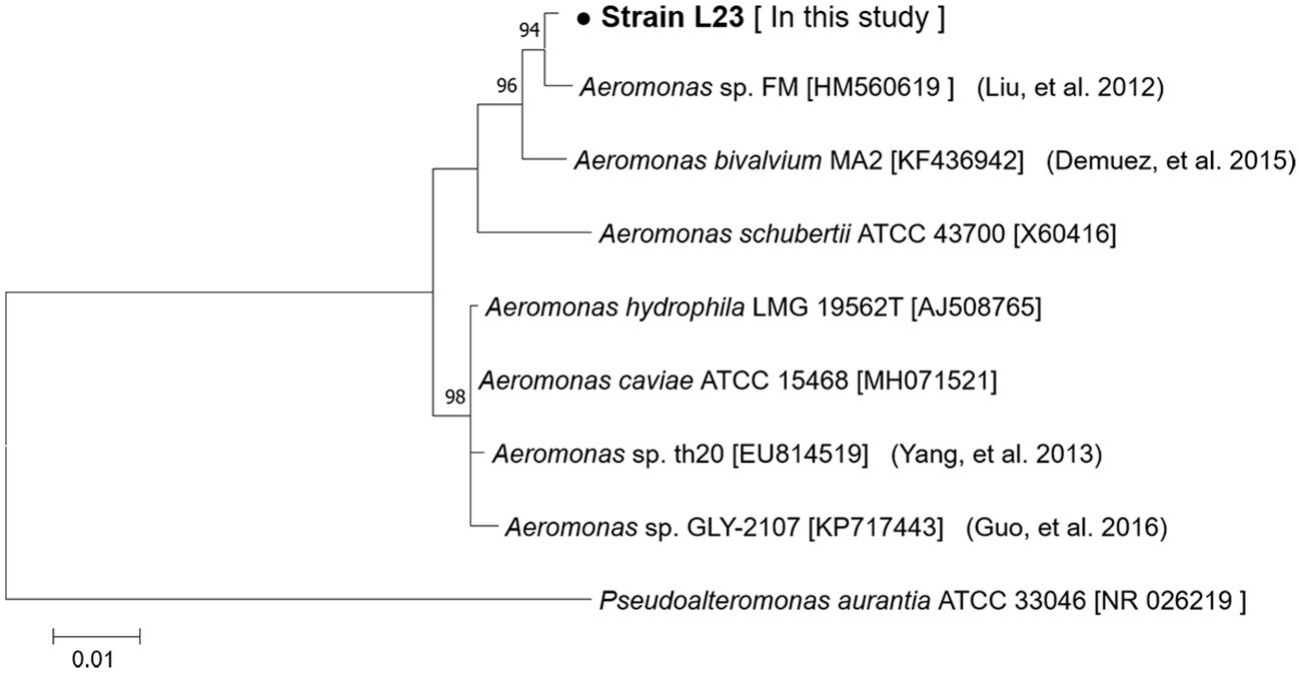
Phylogenetic tree based on the 16S rRNA gene of strain L23. Neighbour-joining phylogenetic tree showing the position of the strain L23 in relation to other closely related species, including previously reported *Aeromonas* sp. Accession numbers correspond to partial 16s rRNA gene sequence. Numerical tree values represent bootstrap support. Scale bar represents expected changes per site.

Among the 36 bacterial strains isolated from the Maiji Lake, strain L23 was selected for further study as it showed the strongest algicidal activity against the target cyanobacteria and green algae. The algicidal activities of this strain against *M*. *aeruginosa* UTEX LB 2385, *M*. *aeruginosa* NHSB, *A*. *variabilis* AG10064, *S*. *quadricauda* AG10003, and *C*. *vulgaris* AG10034 were 88 ± 1.2%, 94 ± 2.6%, 93 ± 0.5%, 82 ± 1.1%, and 47 ± 0.9%, respectively (Fig 1a). The activity began from the first day of the treatment and the rate increased steadily, reaching the maximum after five days (S1 Fig). The cell densities of strain L23 were not significant difference in all mixed culture over time (Fig 1b). The phylogenetic analysis clustered strain L23 with the genus *Aeromonas* (Fig 2) sharing 99.9% similarity of the 16S rRNA gene with *A*. *salmonicida* ATCC 33658 (NR119042). Several studies reported the algicidal activity of *Aeromonas* species isolated from a wetland [8] and a lake [11], which showed algicidal activity against either cyanobacteria or green algae [20]. *Aeromonas* sp. strain FM showed strong algicidal activity against cyanobacterial strains [20] and *A*. *hydrophila* strain AD9 showed high algicidal activity against green algae [8]. The strain L23 of this study showed a broad algicidal spectrum, with stronger activity against all target cyanobacteria than green algae. Interestingly, this strain showed significantly different algicidal activity against the two closely related strains of *M*. *aeruginosa*. The *Microcystis* strains producing the toxins have been reported to show better fitness against environmental stress such as higher oxygen radical concentration, and lower levels of nitrate and phosphate, compared to the strains producing no toxins [21]. Most previous research focused on the algicidal activity of bacteria against target cyanobacteria or green algae. Among numerous studies, *Exiguobacterium* sp. belonging to phylum Firmicutes is one of the rare examples that exhibited algicidal activity against a broad range of algae, including both cyanobacteria and green algae. However, it elicited little algicidal activity against the green algal strains [22]. In contrast, the strain L23 of this study is reported for the first time to be a bacterium belonging to the genus *Aeromonas* that has strong algicidal activity against both cyanobacteria and green algae. The identification of a novel bacterial strain with algicidal activities against a broad range of algal species advances our knowledge of how to control HABs in natural water resources.

### Algicidal mode

**Fig 3:**
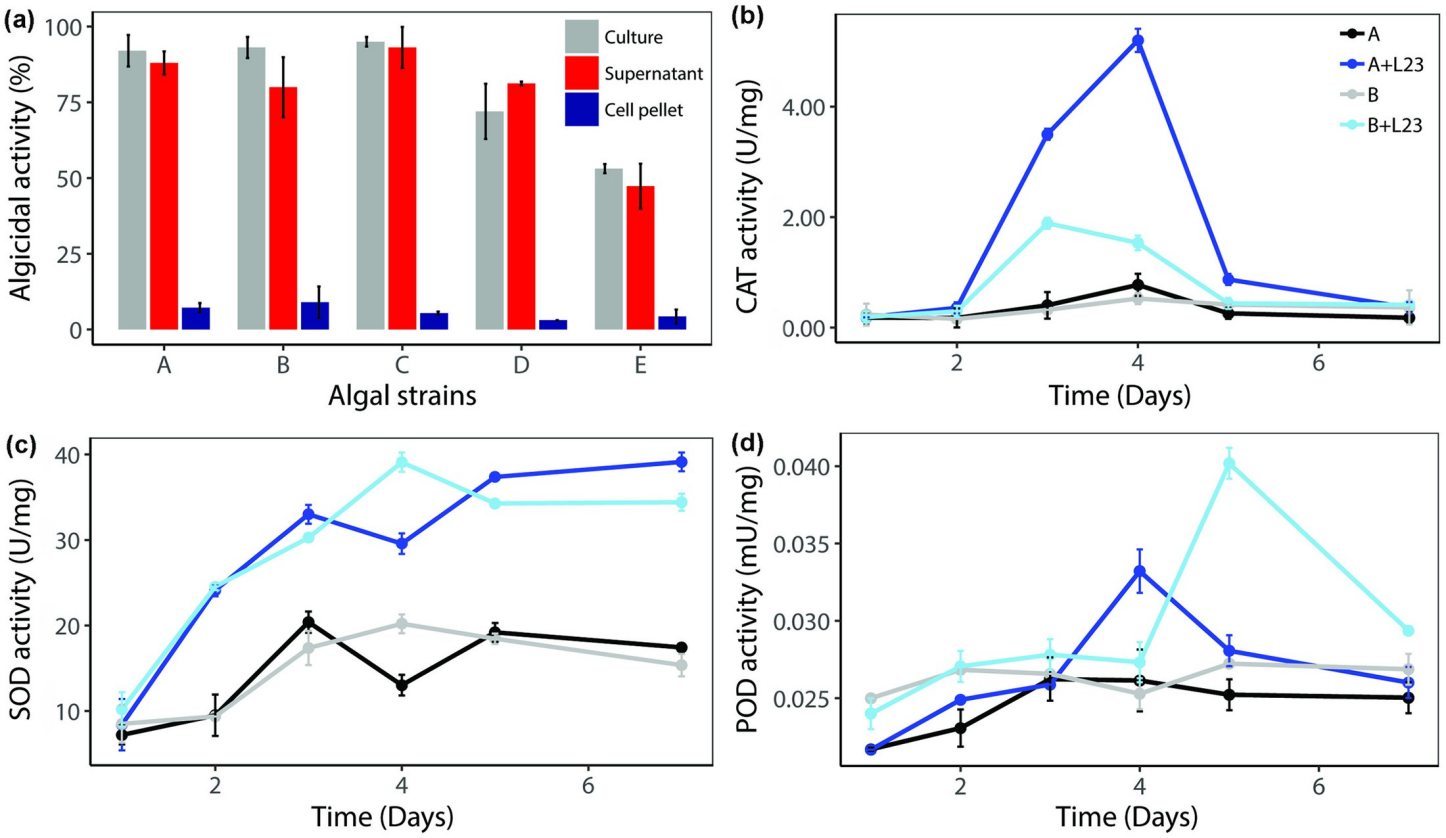
Algicidal mode and mechanism of the strain L23. Algicidal efficiencies of different fractions of the strain L23 culture against (A) *Microcystis aeruginosa* UTEX LB 2385, (B) *Microcystis aeruginosa* NHSB, (C) *Anabaena variabilis* AG10064, (D) *Scenedesmas quadricauda* AG10003, (E) *Chlorella vulgaris* AG10034 after five days of treatment (a). Change in the antioxidant system through the activities of CAT (b), SOD (c), POD (d) of *M*. *aeruginosa* UTEX LB 2385 and *M*. *aeruginosa* NHSB with the treatment of strain L23. Algal culture without the treatment of the strain L23 used as a control. Average values ± standard deviation (n = 3).

The algicidal activities of the strain L23 cell cultureas well as the cell-free supernatant investigated individually. The algicidal activities of the bacterial cell culture and cell-free supernatant were 80%–95% and 78%–93%, respectively, while the bacterial cell pellet showed much lower activity of 3%–9% (Fig 3a). Although the algicidal effect of the bacterial culture toward *C*. *vulgaris* (54% ± 2.3%) was significantly less than against other cyanobacteria and green algae, the activity remained closely comparable to that presented by the supernatant. Likewise, no improvement in lysing activity (6%) was observed for cell pellets against this target. The significant difference in the algicidal activity by the the bacterial cell pellet and cultureand supernatant suggests that the algicidal activity of the strain L23 is based on indirect action of extracellular substance present in the culture medium. Although the algicidal substances were not characterized in this study, *Aeromonas* was identified to produce 3-benzylpiperazine-2,5-dione, 3-methylindole, and clavulanate [11, 12]. These substances are also reported to accelerate the increase in intracellular reactive oxygen species levels in the microalgae [24–26].

### Antioxidant enzyme systems in algal cells

The antioxidant enzyme systems in the algal cells are activated and employed to scavenge reactive oxygen species and relieve cell damage. To investigate the antioxidant defense response of algal cells induced by strain L23, the changes in enzymatic antioxidants superoxide dismutase (SOD), catalase (CAT), and peroxidase (POD) were measured. The levels of antioxidant defense mechanisms in all algal cultures treated with the strain L23 were markedly higher than in the untreated control. The greatest increases in the antioxidant enzyme were shown by both *M*. *aeruginosa* strains (Fig 3a). In this culture, the activities of CAT (Fig 3b) and SOD (Fig 3c) increased significantly, reaching maximum values of 5.0 and 35 U mg^-1^ respectively, at around the day four. After this point, the SOD activity was maintained, whereas the CAT activity showed a rapid drop, converging toward the control level on the day six. Similar to the SOD and CAT activities, the POD activity was also higher in the treatment groups than in the control (Fig 3d), reaching the maximum after four and five days for *M*. *aeruginosa* UTEX LB 2385 and *M*. *aeruginosa* NHSB, respectively. These results revealed that the antioxidant system in the algal cells was expressed in response to oxidative stress caused by the extracellular substance produced by the strain L23. The rapid decrease in CAT is likely to be caused by the death of algae by constant exposure to oxidative agents rather than recovery from or tolerance to them. These results correspond with those of a previous study in which extracellular substances of *Aeromonas* sp. strain FM led to a sharp increase in SOD of *M*. *aeruginosa* culture, but eventually caused breakdown of the antioxidant defense of the algae [27]. In other words, if the oxidative stress of the algicidal substance is not maintained at a sufficient level, the algae may recover to produce a substantial amount of antioxidant enzymes [28, 29], breaking the subtle ecological equilibrium of alga–bacterium interaction, resulting in HAB.

### Algicidal activities vary in different nutrient-rich conditions

**Fig 4:**
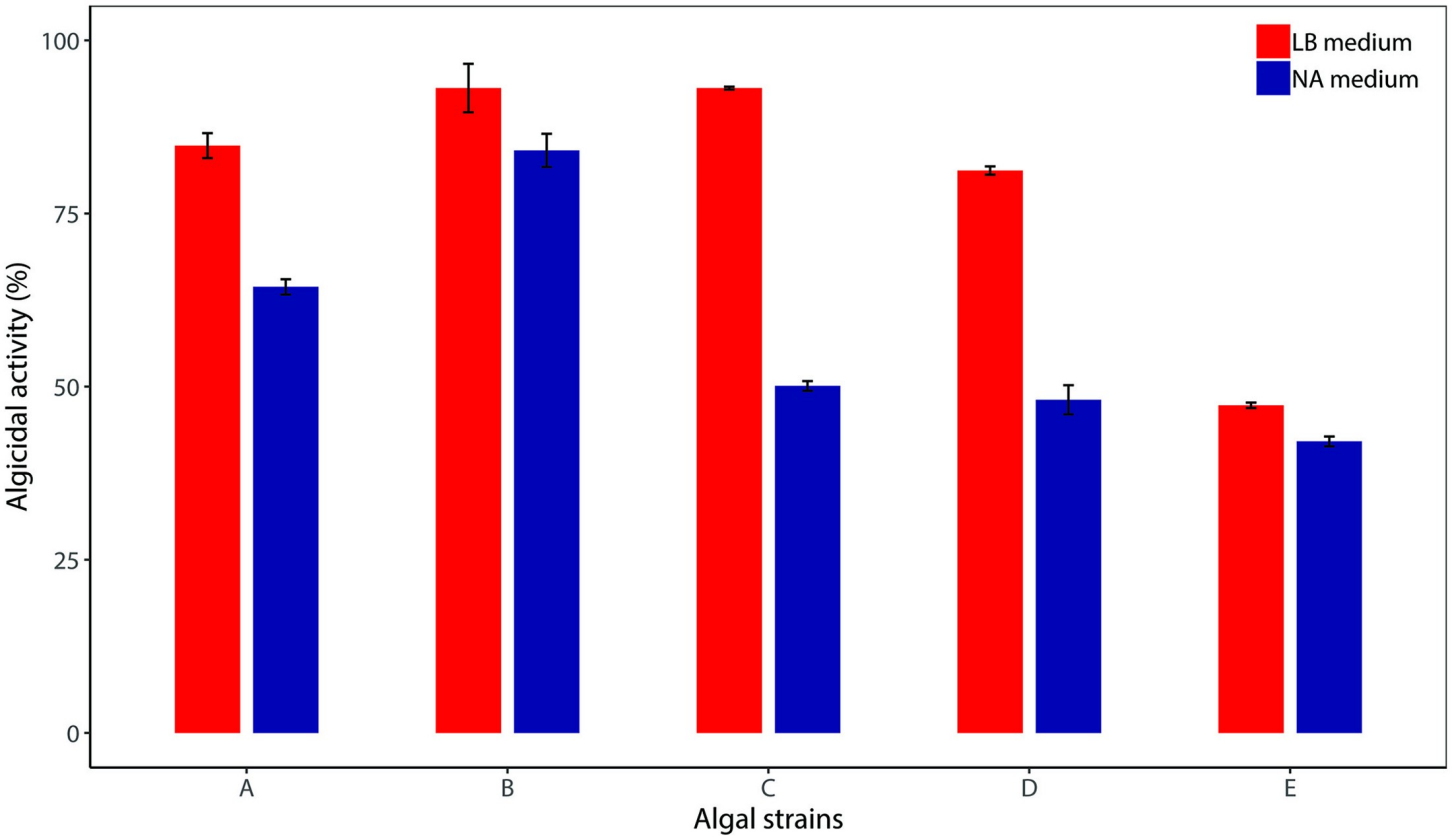
Effect of LB and NB medium on the algicidal activity by the strain L23 culture. Algicidal activities against (A) *Microcystis aeruginosa* UTEX LB 2385, (B) *Microcystis aeruginosa* NHSB, (C) *Anabaena variabilis* AG10064, (D) *Scenedesmas quadricauda* AG10003, (E) *Chlorella vulgaris* AG10034.

The nutritional status of each HAB-susceptible body of water is unique and closely related to the algicidal activity. Despite the importance of the nutritional status in eutrophication, most previous studies focused on the algicidal activity of a single bacterial strain against selected algae in a medium designed to yield the best result [1]. We compared the algicidal activity of the strain L23 grown in two nutrient-rich media (LB and NB) reflecting different eutrophication conditions (Fig 4). The algicidal activity of the strain L23 against the five target algal strains varied from 47% to 94% when grown in LB medium, whereas its activity ranged from 42% to 84% in the NB medium. The LB medium led to 5% to 40% higher algicidal activity against all tested algae compared to the NB medium. The greatest difference (40%) in algicidal rate was observed in *A*. *variabilis* AG10064. The most distinctive of the two medium components is the extract (yeast extract in LB and beef extract in NB). Yeast extract is a rich source of vitamins, a major dietary micronutrient, enhancing the bacterial growth and synthesis of metabolites and extracellular substances. Though the growth rate of strain L23 was higher in the LB medium than the NA medium, same initial cell densities (7 x 10^6^ CFUs mL^-1^) from both media were added in each algal culture to compare the effects on the media on the algicidal activity. The cell densities were not significant varied in the cultures with all algal species. There was a negligible effects of both media indicating that components of the media has no direct effects on algicidal activity (S2 Fig). Zhang and colleagues reported that cell density is the most critical factor to decide the yield of algicidal substances [30]. This indicates that the cell density mediated by different medium types could strongly influence the amount of extracellular substance as well as the algicidal rate. However, the differences of algicidal activities within the same algal species remain unclear. Both yielded similar algicidal rates against *M*. *aeruginosa* UTEX LB 2385, *M*. *aeruginosa* NHSB, and *C*. *vulgaris*, whereas significant decreases in algicidal rate were found against *A*. *variabilis* and *S*. *quadricauda* in the NB medium. The production and composition of extracellular substances were reported to be strongly influenced by substrate type, operation conditions, growth stage, and solution chemistry [31]. Thus, NB medium may be lacking in some vital components required for the production of the extracellular substances that inhibit the growth of specific algae. These results indicated that the algicidal activities might change according to changes in nutritional status.

### Algicidal activity response to nutritional status

**Fig 5:**
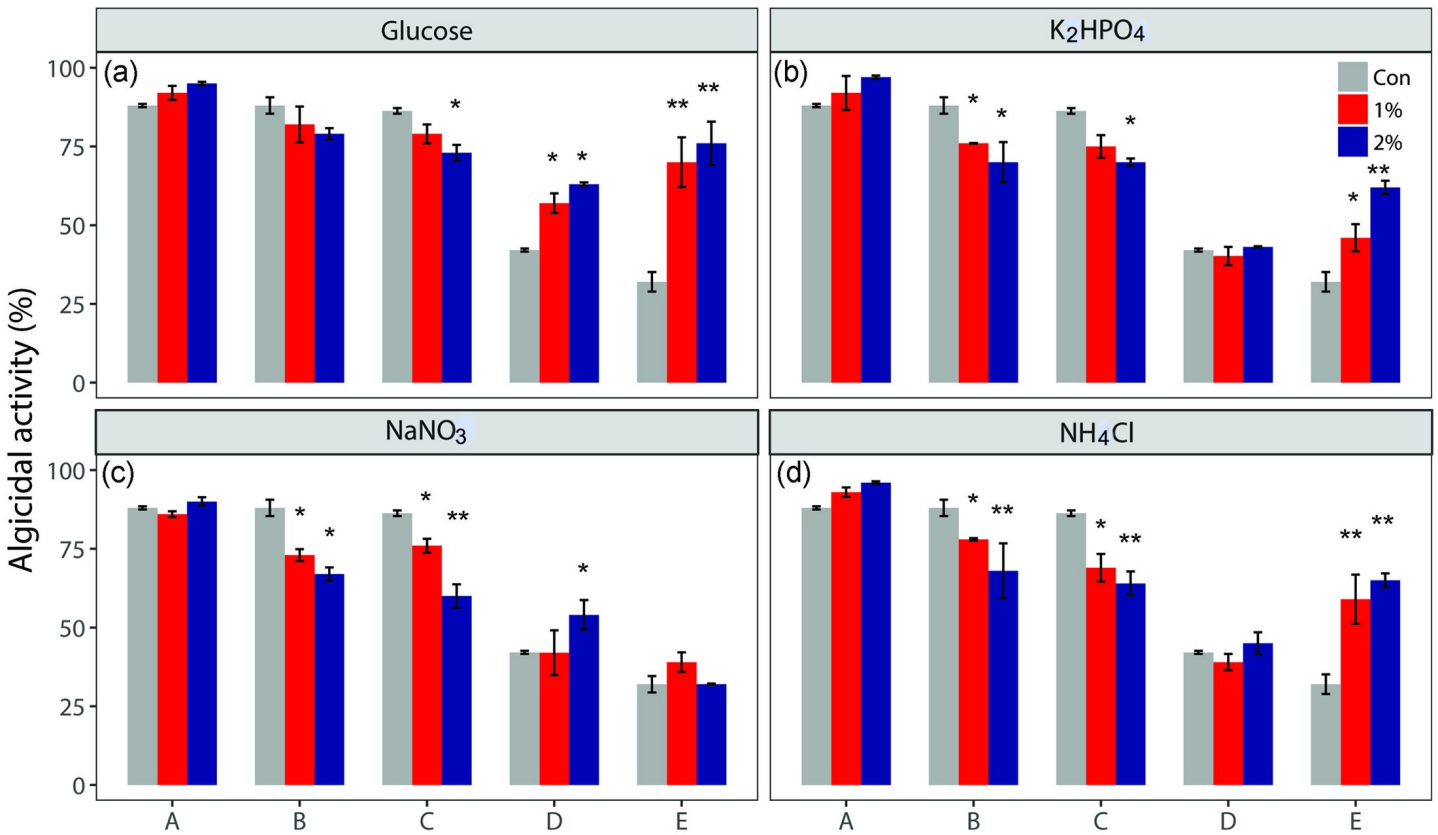
The effect of the nutritional status on the algicidal activity of the strain L23. Algicidal rate of the strain L23 against (A) *Microcystis aeruginosa* UTEX LB 2385, (B) *Microcystis aeruginosa* NHSB, (C) *Anabaena variabilis* AG10064, (D) *Scenedesmas quadricauda* AG10003, (E) *Chlorella vulgaris* AG10034 with the additional dose (one and two percent) of glucose, K_2_HPO_4_, NaNO_3_ and NH_4_Cl compared to control.

The effect of nutritional status on the algicidal activity of the strain L23 is shown (Fig 5). The nutritional status affected the growth of cyanobacteria and green algae differently. When a 1%–2% dosage of all nutrients (glucose, NaNO_3_, NH_4_Cl, and KH_2_PO_4_) was applied, algicidal activity against *M*. *arguinosa* UTEX LB 2385 was nearly unaffected or slightly increased compared with that in the control, while the algicidal activity in the other cyanobacteria decreased significantly (10–20%). In addition, the dosage of the nitrogen and phosphorus sources produced lower algicidal activity than adding the same dosage of the carbon source into the culture. Unlike the algicidal effects on the cyanobacteria, the algicidal activity toward the green algae (*C*. *vulgaris* and *S*. *quadricauda*) increased after the nutrient treatments with glucose, NH_4_Cl, and K_2_HPO_4_. The nutritional condition had a greater impact on *C*. *vulgaris* (10%–50%) than on *S*. *quadricauda* (5%–20%) and the inhibition efficiency of the higher dosage (2%) was greater than that of the lower dosage (1%). These results are consistent with previous results describing that the algicidal activity varied according to the types and concentrations of nitrogen sources, carbon sources, and inorganic salts [1]. The opposite effects of nutritional conditions on the removal of the cyanobacteria and green algae indicated that the algicidal activity seemed to differ in response to nutritional status according to the algal type. There was no significant density variation of bacterial cell in the algal-bacterial mixed culture with additional glucose, NaNO_3_, NH_4_Cl, and KH_2_PO_4_ (S3 Fig). Variation in algicidal activity indicates that the additional nutrients can causes growth variation of algal cells as mentioned in previous studies [32–34] or shift the capacity of producing extracellular substance by *Aeromonas* sp L23. Although the associations between the algicidal substances and algal activities were unclear, the results revealed that the competitive interaction between strain L23 and the tested algal species varied in response to the nutritional statuses. The maintenance of fitness and survival in a changing and stressful environment is mediated through acclimation, which induces short-term change in the performance of an individual that results from phenotypic plasticity [35]. All of the mechanisms that are associated with acclimation limit the amount of metabolic energy that is available for sustaining life. Eutrophic conditions, which are consistent with the conditions after nutrient injection, are likely to have positive effects on the energy status of cyanobacteria. The resource competition modeling between cyanobacteria and green algae support that the cyanobacteria was competitively superior to the green algae at high nutrient level [36]. Based on the physiological responses of cyanobacteria to nutrient loading, for example, the antioxidant enzymes increase slightly or are maintained for 9 days [34]. In contrast, as the nutrients in green algae increase, the efficiency of nitrogen and phosphorus uptake in dark conditions drops rapidly concurrently with no increase of the biomass, and the antioxidant enzymes in the cell also gradually become inactivated [32, 33]. Taking these findings together, a better nutritional status presumably enhances the ability of cyanobacteria to compensate for the negative effects of algicidal activity from the strain L23, which is not the case for the green algae of this study. The phenomenon that only highly tolerant species benefit from the positive aspects of nutrient load can explain why cyanobacteria dominate over green algae in eutrophic conditions.

## Conclusion

This study demonstrated the ability of the *Aeromonas* strain L23, isolated from a lake at which HABs frequently occur, to inhibit the growth of different types of microalgae including three cyanobacteria and two green algae using their extracellular substances in a manner dependent on the nutritional status. Specifically, the algicidal activity for different types of microalgae varied depending on the nutrient loading. It becomes clear that alga-bacterium interactions are highly complex as the interactions between algal species and bacteria varies continuously with nutritional status in the field. These insights will now pave the way toward elaborate studies which contribute a better understanding of the role of algicidal bacteria within algal communities in the field.

## Supporting information

S1 Fig Growth inhibition of cyanobacteria and green algal strains by the strain L23 up to seven days after the treatment. (TIF)

S2 Fig Algicidal effect by LB and NB media in culture of (A) *Microcystis aeruginosa* UTEX LB 2385, (B) *Microcystis aeruginosa* NHSB, (C) *Anabaena variabilis* AG10064, (D) *Scenedesmas quadricauda* AG10003, (E) *Chlorella vulgaris* AG10034. (TIF)

S3 Fig Density variation of the *Aeromonas* sp. L23 in the culture of (A) *Microcystis aeruginosa* UTEX LB 2385, (B) *Microcystis aeruginosa* NHSB, (C) *Anabaena variabilis* AG10064, (D) *Scenedesmas quadricauda* AG10003, (E) *Chlorella vulgaris* AG10034 with the additional dose (one and two percent) of glucose, K_2_HPO_4_, NaNO_3_ and NH_4_Cl compared to control. (TIF)

S1 Dataset (XLSX)
