# Supplementary figures and images for: Nutritional status regulates algicidal activity of Aeromonas sp. L23 against cyanobacteria and green algae
Source: PLoS One. 2019 Mar 12;14(3):e0213370. doi: 10.1371/journal.pone.0213370 (PMC6413897; doi:10.1371/journal.pone.0213370)

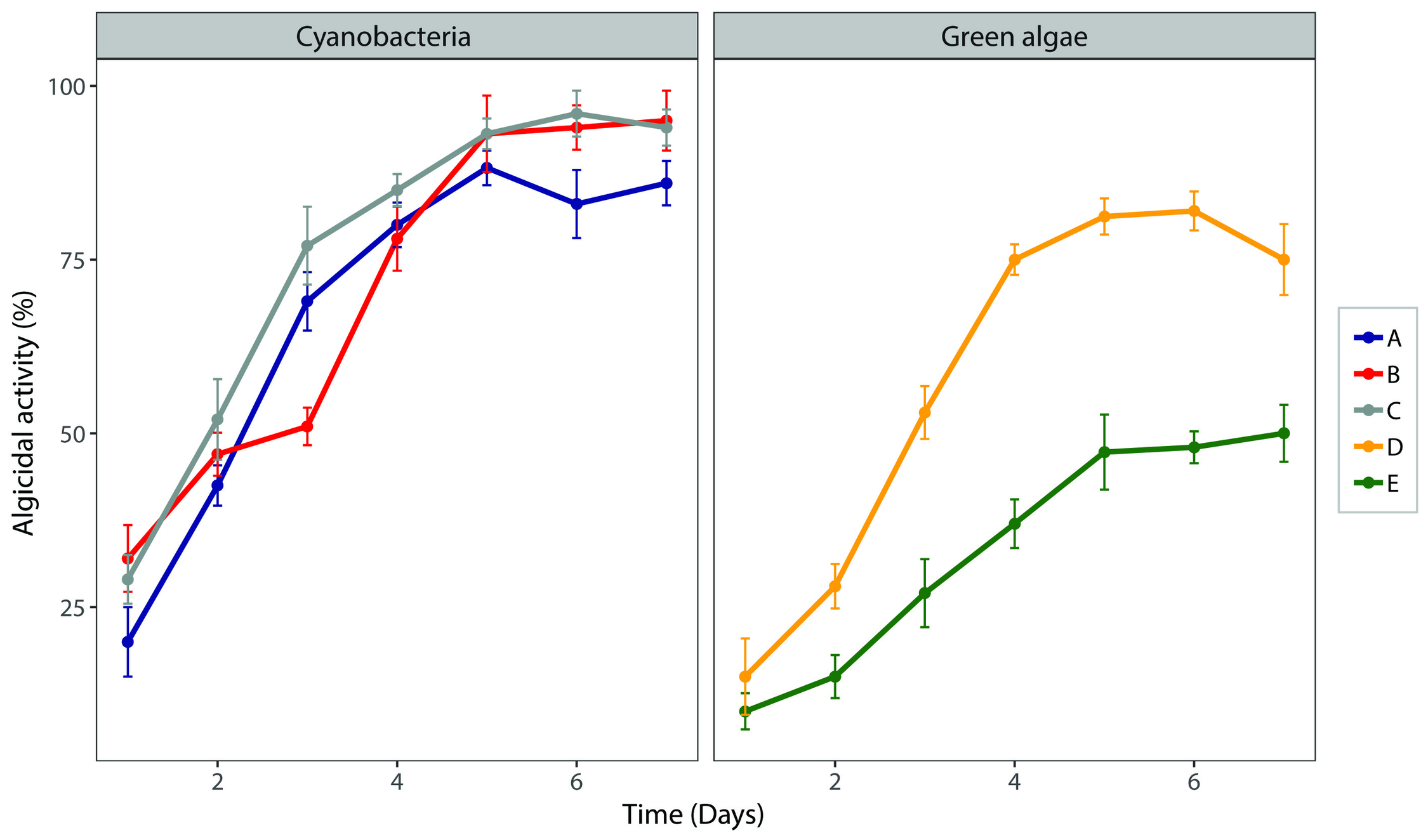

Supplement: S1 Fig — (TIF) [file pone.0213370.s001.tif]

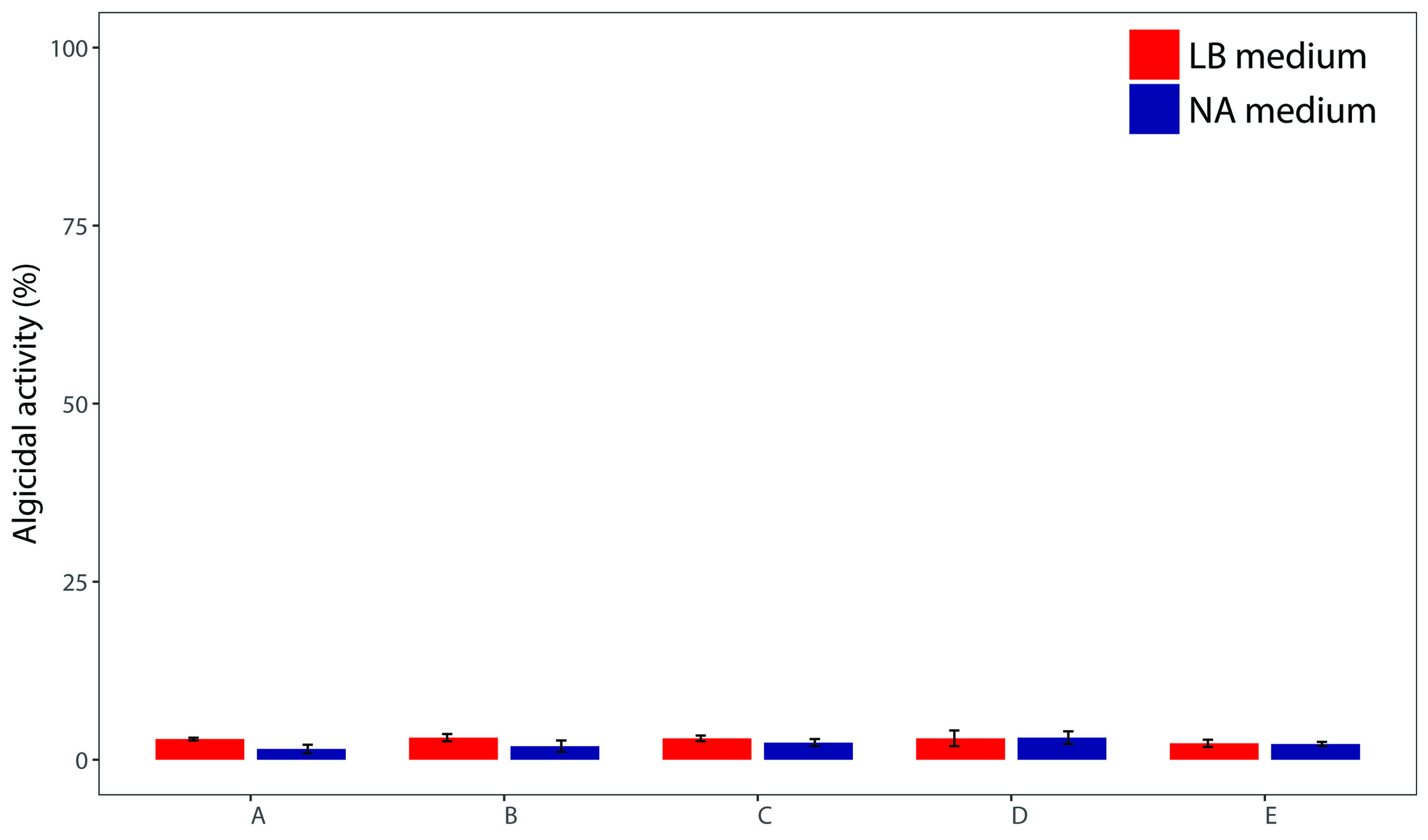

Supplement: S2 Fig — (TIF) [file pone.0213370.s002.tif]

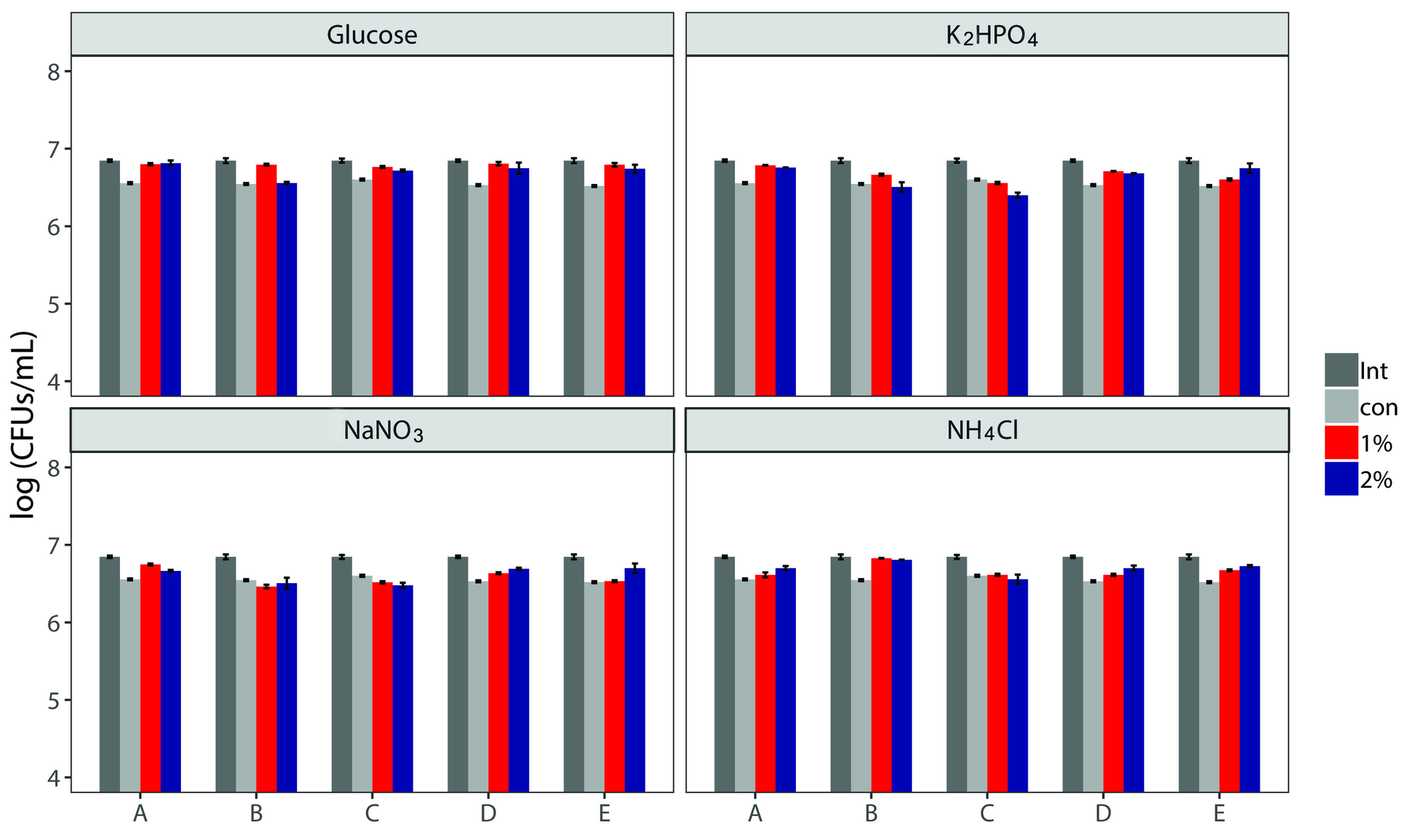

Supplement: S3 Fig — (TIF) [file pone.0213370.s003.tif]
